# Novel Dihydroorotate Dehydrogenase Inhibitors with Potent Interferon-Independent Antiviral Activity against Mammarenaviruses In Vitro

**DOI:** 10.3390/v12080821

**Published:** 2020-07-29

**Authors:** Yu-Jin Kim, Beatrice Cubitt, Yingyun Cai, Jens H. Kuhn, Daniel Vitt, Hella Kohlhof, Juan C. de la Torre

**Affiliations:** 1 Department of Immunology and Microbiology, The Scripps Research Institute, La Jolla, CA 92037, USA; yujin@scripps.edu (Y.-J.K.); bcubitt@scripps.edu (B.C.); 2 Integrated Research Facility at Fort Detrick (IRF-Frederick), National Institute of Allergy and Infectious Diseases (NIAID), National Institutes of Health (NIH), B-8200 Research Plaza, Fort Detrick, MD 21702, USA; caiy@niaid.nih.gov (Y.C.); kuhnjens@niaid.nih.gov (J.H.K.); 3 Immunic Therapeutics, New York City, NY 10036, USA; daniel.vitt@imux.com (D.V.); hella.kohlhof@imux.com (H.K.)

**Keywords:** mammarenavirus, DHODH, pyrimidine biosynthesis, interferon, antiviral

## Abstract

Mammarenaviruses cause chronic infections in rodents, which are their predominant natural hosts. Human infection with some of these viruses causes high-consequence disease, posing significant issues in public health. Currently, no FDA-licensed mammarenavirus vaccines are available, and anti-mammarenavirus drugs are limited to an off-label use of ribavirin, which is only partially efficacious and associated with severe side effects. Dihydroorotate dehydrogenase (DHODH) inhibitors, which block *de novo* pyrimidine biosynthesis, have antiviral activity against viruses from different families, including *Arenaviridae*, the taxonomic home of mammarenaviruses. Here, we evaluate five novel DHODH inhibitors for their antiviral activity against mammarenaviruses. All tested DHODH inhibitors were potently active against lymphocytic choriomeningitis virus (LCMV) (half-maximal effective concentrations [EC_50_] in the low nanomolar range, selectivity index [SI] > 1000). The tested DHODH inhibitors did not affect virion cell entry or budding, but rather interfered with viral RNA synthesis. This interference resulted in a potent interferon-independent inhibition of mammarenavirus multiplication in vitro, including the highly virulent Lassa and Junín viruses.

## 1. Introduction

Mammarenaviruses (*Bunyavirales*: *Arenaviridae*: *Mammarenavirus*) cause chronic asymptomatic infections in their natural reservoir hosts, which are predominantly muroid rodents. Some of these viruses are public health threats because they can be transmitted to humans through aerosol transmission or by direct contact of abraded skin with contaminated material [1]. Several mammarenaviruses, chiefly Lassa virus (LASV) in Western Africa and Junín virus (JUNV) in the Argentinian Pampas, can cause severe disease in humans (Lassa fever and Argentinian hemorrhagic fever, respectively), and pose important public health problems in their endemic regions. LASV is highly prevalent in Western Africa, where the virus infects hundreds of thousands of individuals yearly resulting in a high number of Lassa fever cases that are associated with high morbidity and a high case fatality rate among hospitalized patients [2]. Notably, increased travel has resulted in imported cases of Lassa fever into non-endemic metropolitan regions, including the United States (U.S.) [3,4]. Evidence indicates that LASV-endemic regions are expanding [5], and the identification of novel zoonotic mammarenaviruses, such as Lujo virus (LUJV), responsible for a cluster of severe infections in humans in Southern Africa in 2009 [6], has raised concerns about the emergence of novel arenaviruse hemorrhagic fevers. In addition to JUNV, several other New World mammarenaviruses are responsible for small recurrent outbreaks of high-consequence diseases in humans in South America, including Chapare virus (CHAPV) and Machupo virus (MACV) in Bolivia, Guanarito virus (GTOV) in Venezuela, and Sabiá virus (SBAV) in Brazil [7,8,9,10,11]. Moreover, mounting evidence suggests that the widely distributed lymphocytic choriomeningitis virus (LCMV) may be a neglected human pathogen, contributing to cerebral abnormalities associated with congenital infections [12,13,14,15], and LCMV poses a particular threat to immunocompromised individuals, as illustrated by fatal cases of LCMV infection associated with organ transplants [16].

The JUNV Candid#1 strain is largely considered a safe and effective live attenuated vaccine to prevent cases of Argentinian hemorrhagic fever [17] and was licensed in 2006 by the regulatory agency of Argentina for use exclusively in Argentina. In the U.S., Candid#1 is considered an investigational new drug (IND). No FDA-licensed mammarenavirus vaccines are available, and current anti-mammarenavirus therapy is limited to an off-label use of ribavirin, which is only partially efficacious and associated with severe side effects [18,19,20]. Several small molecules, including the broad-spectrum mammarenavirus RNA-directed RNA polymerase inhibitor favipiravir and the mammarenavirus glycoprotein GP2-mediated fusion inhibitor ST-193, have promising anti-mammarenaviral effects in various animal models of mammarenavirus-induced human diseases [21,22,23,24]. Furthermore, recent drug repurposing strategies have identified different types of anti-mammarenaviral compounds, targeting distinct host cell factors and pathways, including *de novo* pyrimidine biosynthesis [25]. Inhibitors of *de novo* pyrimidine biosynthesis have broad-spectrum antiviral activity [25,26,27,28,29]. Dihydroorotate dehydrogenase (DHODH) is a key enzyme involved in de novo pyrimidine biosynthesis, converting dihydroorotate (DHO) into orotate, and DHODH has been validated as one of the targets of pyrimidine biosynthesis inhibitors exhibiting antiviral activity [30]. We investigated the antiviral activity of a series of novel DHODH inhibitors (i.e., Compound (Cmp) 1, Cmp 2, Cmp 3, Cmp 4, and Cmp 5) against diverse mammarenaviruses. Cmp 1, Cmp 2, Cmp 3, and Cmp 5 are new molecular entities with undisclosed structures. In addition to their inhibitory effect on DHODH, these compounds were also active against retinoic acid-related orphan receptor C (RORC, also known as RORg) with half-maximal inhibitory concentration (IC_50_) values ranging from 5 to 50 nM. Cmp 4 is IM90838 (IMU-838), the Ca^2+^ salt of vidofludimus (Figure 8A), which is currently under testing in phase 2b clinical trials against multiple sclerosis, ulcerative colitis, and primary sclerosing cholangitis. Both vidofludimus and Cmp 4 depend on the same active ingredient in blood (vidofludimus) for their mechanism of action, toxicology, and pharmacology [31].

All DHODH inhibitors tested in this study had an IC_50_ lower than 1 µM for human DHODH (hDHODH) and were potently active against LCMV (half-maximal effective concentrations [EC_50_] in the low nanomolar range, selectivity index [SI] > 1000). Importantly, these inhibitors were also active against LASV and JUNV. The DHODH inhibitors did not affect virus cell entry or budding, but rather targeted replication and transcription of the mammarenavirus genome, i.e., the biosynthetic processes directed by the mammarenavirus ribonucleoprotein complex (vRNP). The antiviral activity of some DHODH inhibitors has been linked to the activation of the type I interferon (IFN-I) system and the expression of IFN-stimulated genes (ISGs) [32]. However, the novel DHODH inhibitors exhibited potent IFN-I-independent anti-mammarenaviral activity. Non-limiting exogenous uridine supply promoted the pyrimidine salvage pathway and restored normal levels of virus multiplication in the presence of DHODH inhibitors, which could be partially prevented by inhibiting uridine-cytidine kinase 2 (UCK2), a key enzyme of the pyrimidine salvage pathway. The inhibition of DHODH in combination with UCK2 inhibitors might open a new avenue for combination therapy to target rapidly replicating RNA viruses, including human pathogenic mammarenaviruses.

## 2. Materials and Methods

### 2.1. Cells and Viruses

Grivet (*Chlorocebus aethiops*) Vero E6 (ATCC CRL-1586) and Homo sapiens A549 (ATCC CCL-185) and 293T (ATCC CRL-3216) cell lines were maintained in Dulbecco’s modified Eagle’s medium (DMEM) (ThermoFisher Scientific, Waltham, MA, USA) containing 10% heat-inactivated fetal bovine serum (FBS), 2 mM of l-glutamine, 100 µg/mL of streptomycin, and 100 U/mL of penicillin. The tri-segmented form of the live attenuated vaccine Candid#1 strain of JUNV expressing green fluorescent protein (GFP, r3Can/GFP) [33], recombinant LCMV expressing *Zoanthus* sp. green fluorescent protein (ZsG) fused to nucleoprotein (NP) via a P2A ribosomal skipping sequence (rLCMV/ZsG-P2A-NP, referred to as rLCMV/ZsG) [34], a single-cycle infectious rLCMV expressing ZsG (rLCMV∆GPC/ZsG-P2A-NP, here referred to as rLCMV∆GPC/ZsG) [34], an immunosuppressive strain of LCMV, clone 13 (CL-13) expressing ZsG (rCL-13/ZsG), and an LASV-expressing GFP (rLASV/GFP) [35] have been described previously.

### 2.2. Compounds

The *in vitro* inhibition of hDHODH was measured using an N-terminally truncated recombinant hDHODH enzyme as described previously [36]. Briefly, the hDHODH concentration was adjusted in a way that an average slope of approximately 0.2 AU/min served as the positive control (e.g., without inhibitor). The standard assay mixture contained 60 µM 2,6-dichloroindophenol (Sigma, St. Louis, MO, USA), 50 µM decylubiquinone (Sigma), and 100 µM dihydroorotate (Sigma). The hDHODH enzyme with or without at least six different concentrations of the compounds was added, and measurements were performed in 50 mM TrisHCl, 150 mM potassium chloride (KCl) (Merck), and 0.1% Triton X-100 (Sigma) at pH 8.0 and at 30 °C [37]. The reaction was started by adding dihydroorotate and measuring the absorption at 600 nm for 2 min. For the determination of the IC50 values, each data point was recorded in triplicate. DHODH inhibitors Cmp 1, Cmp 2, Cmp 3, Cmp 4, and Cmp 5 were provided by Immunic Therapeutics. Brequinar, uridine, 2′-deoxyuridine, cytidine, and 2′-deoxycytidine were purchased from Sigma. UCK2 inhibitor, UCK2-IN-20874830 was obtained from ChemDiv (San Diego, CA, USA).

### 2.3. Cell Cytotoxicity Assay and Half-Maximal Cytotoxic Concentration (CC_50_) Determination

Cell viability was assessed using CellTiter 96 AQ_ueous_ One Solution reagent and cell proliferation assay (Promega, Madison). This method determined the number of viable cells based on conversion of formazan product from 3-(4,5-dimethylthazol-2-yl)-5-(3-carboxymethoxyphenyl)-2-(4-sulfophenyl)-2H-tetrazolim by nicotinamide adenine dinucleotide phosphate (NADPH) or reduced NADPH (NADH) generated in living cells. A549 cells were plated on 96-well clear-bottom plates (2.0 × 10^4^ cells/well). Serial dilutions (three-fold) of each compound were added to cells and, 48 h after drug treatment, the viability reagent was added and incubated for 15 min (37 °C and 5% carbon dioxide [CO_2_]). Absorbance was measured at 490 nm using an enzyme-linked immunosorbent assay (ELISA) reader (SpectraMax ABS Plus, Molecular Devices, Sunnyvale, CA, USA). The resulting optical densities were normalized with dimethylsulfoxide (DMSO); the vehicle control group was adjusted to 100%. CC_50_ was determined using Prism (GraphPad, San Diego, CA, USA).

### 2.4. Viral Growth Kinetics and EC_50_ Determination

For growth kinetics, virus was added to cells (200 µL/well in a 24-well plate) at the indicated multiplicity of infection (MOI). After 90 min of adsorption, virus inocula were removed, cells were washed twice with DMEM and 2% FBS, and fresh media containing the indicated compounds and concentrations were added. At the indicated hours post-infection (pi), tissue culture supernatants (TCSs) were collected and viral titers were determined using a focus-forming assay (FFA) [38]. For EC_50_ determination, cells were plated on 96-well clear-bottom black plates (2.0 × 10^4^ cells/well) and incubated for 20 h at 37 °C and 5% CO_2_. Cells were pre-treated for 2 h before infection with three-fold serial dilutions of each compound. Cells were infected (MOI = 0.01) with rLCMV/ZsG-P2A-NP in the presence of compounds. At 48 h pi, cells were fixed with 4% paraformaldehyde, nuclei were stained with 4′,6-diamidino-2-phenylindole (DAPI), and ZsG expression was determined by fluorescence using a fluorescent plate reader (Synergy H4 Hybrid Multi-Mode Microplate Reader, BioTek, Winooski, VT, USA). Mean relative fluorescence units were normalized with the vehicle control group (DMSO), which was adjusted to 100%. ZsG expression was normalized for total cell protein in lysate (Pierce BCA Protein Assay Kit, ThermoFisher Scientific). EC_50_ was determined using Prism. SIs for hit compounds were determined using the ratio CC_50_:EC_50_.

### 2.5. Time-of-Addition Assay

A549 cells were seeded (2 × 10^4^ cells/well) on 96-well clear-bottom black plates and incubated for 20 h at 37 °C and 5% CO_2_. Cells were treated with each compound (5 µM) at either 2 h prior to or 2 h following infection. Cells were infected (MOI = 0.5) with single-cycle infectious rLCMV∆GPC/ZsG to prevent the confounding factors caused by multiple rounds of infection. At 48 h pi, cells were fixed with 4% paraformaldehyde and nuclei were stained with DAPI. ZsG expression levels were determined using a fluorescent plate reader (Synergy H4 Hybrid Multi-Mode Microplate Reader, BioTek, Winooski, VT, USA). Mean relative fluorescence units were normalized to the vehicle control (DMSO) group, which was adjusted to 100%.

### 2.6. LCMV Minigenome (MG) Assay

The LCMV MG assay was performed as described [39]. Briefly, 293T cells were cultured on poly-l-lysine-coated 12-well plates (4.5 × 10^5^ cells/well). Cells were transfected using lipofectamine 2000 (2.5 µL/µg of DNA) (ThermoFisher Scientific), with Pol II-based expression plasmids (pCAGGS) for T7 RNA polymerase (pC-T7, 0.5 µg), LCMV (NP) (pC-NP, 0.3 µg), and LCMV RNA-directed RNA polymerase (L) (pC-L, 0.3 µg), together with a plasmid directing intracellular synthesis of an LCMV MG expressing the chloramphenicol acetyl transferase (CAT) reporter gene (pT7-MG/CAT, 0.5 µg). After 5 h, the transfection mixture was replaced with fresh media and incubated for 72 h at 37 °C and 5% CO_2_. At 72 h post-transfection, whole-cell lysates (WCLs) were harvested to determine expression levels of CAT using a CAT ELISA kit (Roche, Sydney, Australia). Briefly, WCLs were prepared with 0.5 mL of lysis buffer, and 10 µL of each sample were used for the reaction. Diluted samples were added onto CAT-ELISA plates and incubated for 1 h at 37 °C. After incubation with samples, plates were washed, and primary antibody (anti-CAT-digoxigenin) and secondary antibody (anti-CAT-digoxigenin-peroxidase) were added sequentially, followed by the substrate. After 20 min, absorbance was measured using the ELISA reader at 405 nm for samples and 490 nm for the reference.

### 2.7. Budding Assay

The luciferase-based budding assay was performed as described [40]. Briefly, 293T cells were seeded on poly-L-lysine-coated 12-well plates (3.5 × 10^5^ cells/well). After overnight incubation, 2 µg of DNA of either pC-LASV-Z-Gaussia luciferase (GLuc) or pC-LASV-mutant Z[G2A]-GLuc were transfected using Lipofectamine 2000 (2.5 µL/µg of DNA). After 5 h, transfection mixtures were replaced with fresh media containing the indicated hit compounds. After 48 h, TCSs containing virion-like particles (VLPs) were harvested and clarified by low-speed centrifugation to remove cell debris. Aliquots (20 µL each) from TCS samples were added to 96-well black plates (VWR, West Chester, PA, USA), and 50 µL of SteadyGlo luciferase reagent (Promega) were added to each well. WCLs from the same samples were processed to determine cell-associated activity of GLuc. The luminescence signal was measured using the Berthold Centro LB 960 luminometer (Berthold Technologies, Oak Ridge, TN, USA). The activity (relative light units) of GLuc in TCSs and WCLs was used as a surrogate of Z expression. Budding efficiency was defined as the ratio Z_VLP_:(Z_VLP_+Z_WCL_).

### 2.8. Virus Titration

Virus titers were determined by a focus-forming assay [38]. Serial dilutions of samples (10-fold) were used to infect Vero E6 cell monolayers in 96-well plates (2 × 10^4^ cells/well). At 20 h pi, cells were fixed with 4% paraformaldehyde in phosphate-buffered saline. The foci of cells infected with rLCMV/ZsG, rLCMV∆GPC/ZsG, or rLASV/GFP were determined by the epifluorescence of fluorescent reporter gene expression. The foci of cells infected with wild-type LCMV were identified by rat monoclonal antibody VL4 against NP (Bio X Cell, West Lebanon, NH, USA) conjugated with Alexa Fluor 488.

### 2.9. Reverse Transcription Polymerase Chain Reaction (RT-PCR)

A549 cells were infected with rLCMV/ZsG-P2A-NP in the presence (5 µM) of the indicated compounds or vehicle control (DMSO). Total cellular RNA was isolated using TriReagent (Invitrogen, Carlsbad, CA, USA) according to the manufacturer’s instructions. Total RNA (1~3.5 µg) was reverse-transcribed to cDNA using a SuperScript IV First-Strand Synthesis System (ThermoFisher Scientific). Target sequences were amplified by PCR with primer sets listed in Section 2.10. PCR products were resolved by agarose gel electrophoresis and visualized by ethidium bromide staining.

### 2.10. Primers

- LCMV NP-specific primers
  forward: 5′-ATGCAGTCCATGAGTGCACAGT-3′
  reverse: 5′-GGTGAAGGATGGCCATACATAG-3′
- Glyceraldehyde 3-phosphate dehydrogenase (GAPDH)-specific primers
  forward: 5′-TGACATCAAGAAGGTGGTGAAGCAG-3′
  reverse: 5′-ATTGTCATACCAGGAAATGAGCTTGAC-3′
- IFNB-specific primers
  forward: 5′-TCAGTGTCAGAAGCTCCTGT-3′
  reverse: 5′-ACAGCATCTGCTGGTTGAAG-3′
- Interferon-stimulated gene 15 (ISG15)-specific primers
  forward: 5′-TGAGAGGCAGCGAACTCATCT-3′
  reverse: 5′-AAGGTCAGCCAGAACAGGTCGT-3′
- Interferon-induced protein with tetratricopeptide repeats 1 (IFIT1)-specific primers
  forward: 5′- GCCTTGCTGAAGTGTGGAGGAA-3′
  reverse: 5′-GCTTTTCTCTGTTCTGCCCTCT-3′
- DExD/H-box helicase 58 (DDX58)-specific primers
  forward: 5′-CACCTCAGTTGCTGATGAAGGC-3′
  reverse: 5′- CGGGCACAGAATATCTTTGCTC-3′
- Signal transducer and activator of transcription 1 (STAT1)-specific primers
  forward: 5′-ATGGCAGTCTGGCGGCTGAATT-3′
  reverse: 5′-GATGCACCCATCATTCCAGAGA-3′
- Interferon alpha inducible protein 27 (IFI27)-specific primers
  forward: 5′-TAAGACGGTGAGGTCAGCTTCA-3′
  reverse: 5′-ACCCAATGGAGCCCAGGATGAA-3′
- Interferon regulatory factor 1 (IRF1)-specific primers
  forward: 5′-GAGGAGGTGAAAGACCAGAGCA-3′
  reverse: 5′-CCAGGTTCATTGAGTAGGTACC-3′
- IRF9-specific primers
  forward: 5′-TACTCACTGCTGCTCACCTTCA-3′
  reverse: 5′-AGTCTGCTCCAGCAAGTATCGG-3′
- IFIT2-specific primers
  forward: 5′-GGAGCAGATTCTGAGGCTTTGC-3′
  reverse: 5′-GCAGGACTAACCTCTATGGGAT-3′
- IRF7-specific primers
  forward: 5′-ACCATCTGCTGACAGCGTCAT-3′
  reverse: 5′-GCTGCTATCCAGGGAAGACACA-3′
- C-X-C motif chemokine 10 (CXCL10)-specific primers
  forward: 5′-GGTGAGAAGAGATGTCTGAATCC-3′
  reverse: 5′-GGCAGTGGAAGTCCATGAAGTA-3′
- Interferon-induced GTP-binding protein Mx1 (MX1)-specific primers
  forward: 5′-GGCTGTTTACCAGACTCCGACA-3′
  reverse: 5′-GATCTCCTCCATGGAAGAGTCT-3′

### 2.11. Animal Studies

Adult (6-week-old) C57BL/6 inbred laboratory mice (Scripps Research breeding colony) were inoculated intravenously (IV) with LCMV CL-13 (2 × 10^6^ FFU) and treated with Cmp 4 (150 mg/kg/d) or vehicle control, administered orally. Treatment was administered daily from Day 1 to Day 17 pi. Mice were monitored daily for the development of clinical signs, weight loss, and survival. All animal experiments were conducted under Protocol 09-0137-4, approved by The Scripps Research Institute Institutional Animal Care and Use Committee (IACUC).

### 2.12. Biosafety

All experiments involving the use of rLASV/GFP were performed under maximum containment, biosafety level 4 (BSL-4), conditions in the BSL-4 suites of the National Institutes of Health (NIH) National Institute of Allergy and Infectious Diseases (NIAID) Integrated Research Facility at Fort Detrick (IRF-Frederick) [41] following approved standard operating procedures [42,43]. All other experiments were performed under BSL-2 conditions.

## 3. Results

### 3.1. Effect of DHODH Inhibitors on Mammarenavirus Multiplication

**Figure 1:**
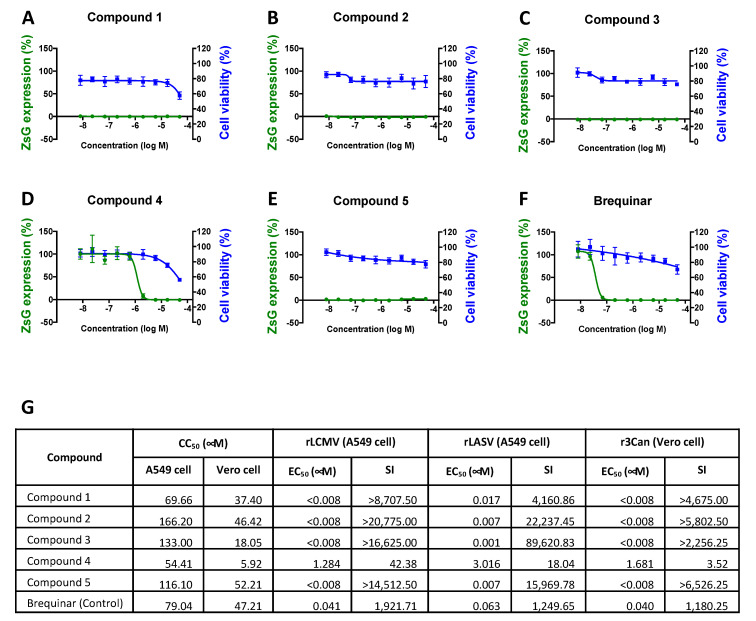
Effect of dihydroorotate dehydrogenase (DHODH) inhibitors on lymphocytic choriomeningitis virus (LCMV) multiplication. (**A**–**F**) Effect on LCMV propagation (green line) and cell viability (blue line) of the indicated compounds. A549 cells were treated with serial dilutions of each compound starting 2 h prior to infection (multiplicity of infection [MOI] = 0.01) with clone 13 (CL-13) expressing *Zoanthus* sp. green fluorescent protein (ZsG) (rCL-13/ZsG). Levels of ZsG expression were determined at 48 h pi (four replicates). Cell viability (six replicates) was evaluated using the CellTiter-Glo assay after 48 h of treatment. Data were normalized with respect to the vehicle control group. Error bars represent mean +/− SEM. (**G**) CC_50_, EC_50_, and selectivity index (SI = CC_50_/EC_50_) values for recombinant LCMV (rLCMV), Lassa virus (rLASV), and r3Can.

We investigated the dose-dependent effects of five novel DHODH inhibitors (i.e., Cmp 1, Cmp 2, Cmp 3, Cmp 4, and Cmp 5) on rLCMV/ZsG multiplication, cell viability, and *in vitro* hDHODH inhibition (Figure 1 and Figure 8). A549 cells were treated with three-fold serial dilutions of each compound. At 2 h post-treatment, cells were infected with rLCMV/ZsG (MOI = 0.01) and at 48 h pi, ZsG expression levels were determined, as described in the Materials and Methods section. Normalized ZsG expression levels were used to determine EC_50_ values. Cell viability was determined by formazan production, and values were normalized for CC_50_ calculation. Brequinar, a known DHODH inhibitor [44,45,46], was used as a control (Figure 1F). All tested DHODH inhibitors also strongly inhibited rLASV/GFP and rCan/GFP multiplication with high SI values (Figure 1G).

**Figure 2:**
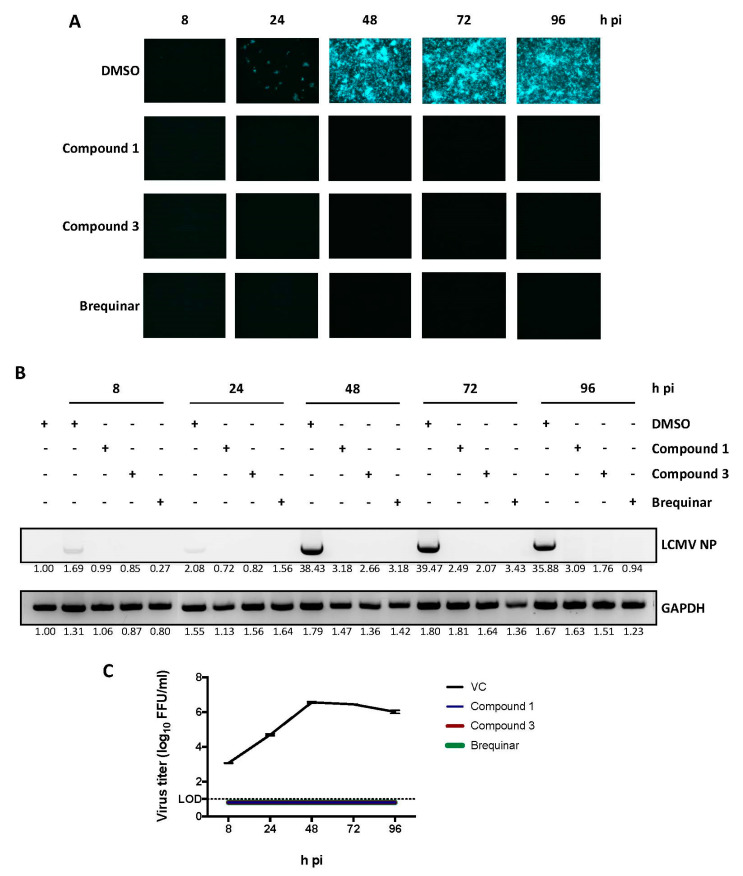
Effect of selected DHODH inhibitors on LCMV multiplication. A549 cells were infected (MOI = 0.01) with rCL-13/ZsG in the presence of the indicated DHODH inhibitors (5 µM) or dimethylsulfoxide (DMSO) vehicle control (VC). (**A**) At the indicated hour (h) post-infection (pi), numbers of ZsG-positive cells were determined by epifluorescence. (**B**) At the indicated time point, total cellular RNA was purified and equal amounts (1 µg) were used in reverse transcription reactions, using random hexamer primers to generate complementary DNAs (cDNAs) that were used in polymerase chain reactions (PCRs) with specific primers to amplify a segment of 644 bp of the viral nucleoprotein (NP) gene. PCR products were resolved by agarose gel electrophoresis and visualized by ethidium bromide staining. (**C**) At the indicated h pi, tissue culture supernatants were harvested, and virus titers were determined. Values for VC-treated samples correspond to two replicates. Error bars represent mean +/− standard error of the mean (SEM). The number below each band indicates the normalized fold change on band intensity compared to mock-infected and vehicle-treated control cells.

To further characterize the antiviral activity of the newly tested DHODH inhibitors, we selected Cmp 1 and Cmp 3 as representative compounds with medium (8.7 × 10^3^) and high (1.6 × 10^4^) SI values, respectively. These compounds were examined for their effects on virus cell-to-cell propagation, viral RNA synthesis, and the production of infectious progeny over time (Figure 2). A549 cells were infected with rLCMV/ZsG (MOI = 0.01) in the presence of each compound (5 µM). At the indicated time points, the number of virus-infected cells (Figure 2A), concentrations of viral RNA (Figure 2B), and the production of infectious viral progeny (Figure 2C) were determined. Both Cmp 1 and Cmp 3 induced strong inhibition of the cell-to-cell propagation of rLCMV/ZsG that correlated with reduced levels of viral RNA synthesis and production of infectious progeny.

### 3.2. Broad-Spectrum Antiviral Effects of DHODH Inhibitors

**Figure 3:**
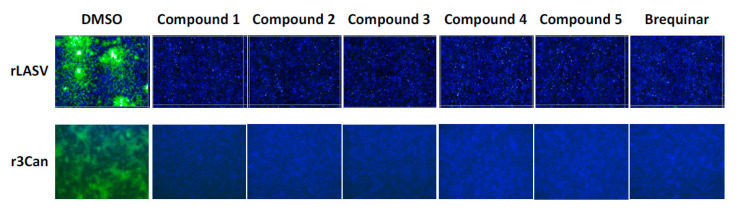
Inhibition of Lassa virus (LASV) and Junín virus (JUNV) multiplication by DHODH inhibitors. A549 cells were infected with rLASV/GFP (MOI = 0.01) and Vero cells were infected with r3Can/GFP (MOI = 0.5) in the presence of the indicated inhibitors (5 µM). At 72 h (for rLASV) or 96 h (for r3Can) pi, cells were fixed, and the numbers of green fluorescent protein (GFP) -positive cells were determined by epifluorescence. Nuclei were visualized by 4′,6-diamidino-2-phenylindole (DAPI) staining.

We examined the antiviral activity of the DHODH inhibitors against two other mammarenaviruses, LASV and JUNV (Figure 3). A549 cells (top row) were exposed (MOI = 0.1) to rLASV/GFP in the presence of each compound (5 µM), and IFN-deficient Vero E6 cells (bottom row) were exposed to the highly attenuated r3Can/GFP (MOI = 0.5). All five novel DHODH inhibitors, as well as brequinar, strongly inhibited rLASV/GFP and r3Can/GFP propagation (Figure 3).

### 3.3. Effect of DHODH Inhibitors on Different Steps of LCMV Lifecycle

**Figure 4:**
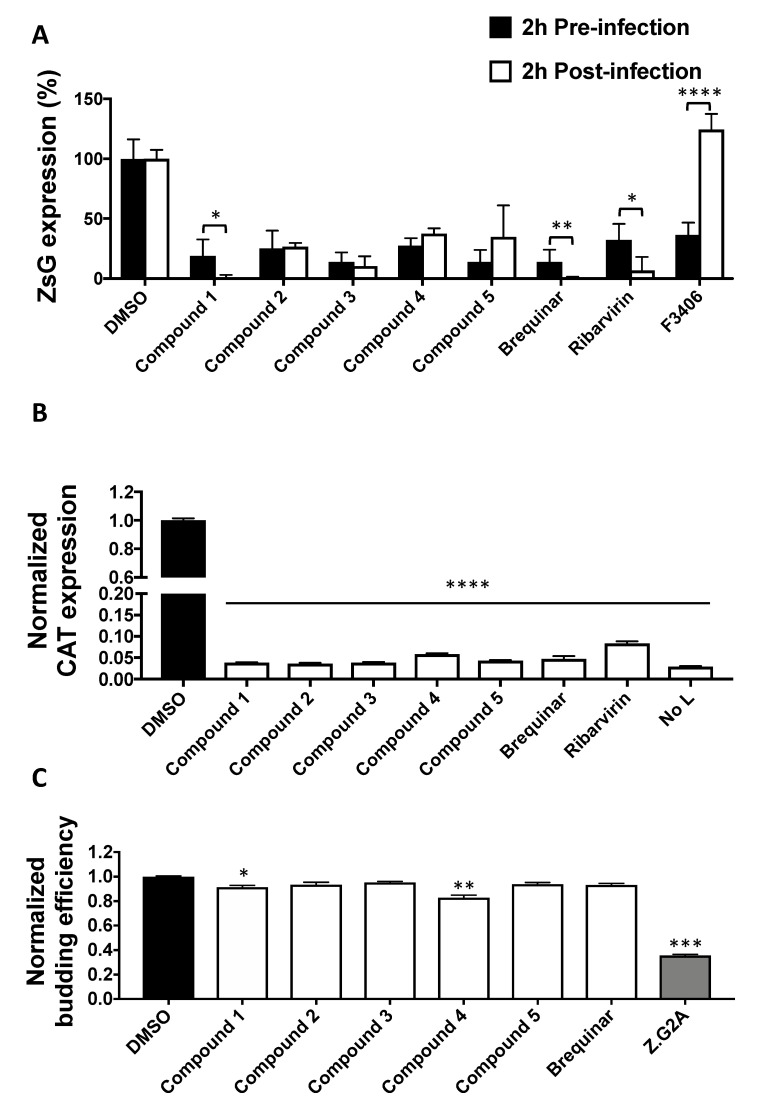
Effect of DHODH inhibitors on different steps of the virus lifecycle. (**A**) Time-of-addition assay. A549 cells were treated with the indicated compounds (5 µM) 2 h before or after infection (MOI = 0.5) with the single-cycle infectious rLCMV∆GPC/ZsG. At 48 h pi, ZsG expression levels were determined. Values were normalized to vehicle control cells. (**B**) Effect of DHODH inhibitors on LCMV minigenome (MG)-derived reporter gene expression. 293T cells were transfected with pC-T7, pMG-chloramphenicol acetyltransferase (CAT), pC-NP, and pC-L. At 5 h after transfection, fresh medium containing the indicated compound (5 µM) was added. Forty-eight hours later, cell lysates were prepared, and CAT expression levels were determined by CAT-ELISA. Values were normalized to the vehicle control group. As a negative control, an empty vector was substituted for pC-L (No L). (**C**) Effect of the indicated compounds on Z-mediated budding. 293T cells were transfected with pC-LASV-Z-GLuc. At 48 h post-transfection, Z budding efficiency (BE) was determined by measuring GLuc activity associated with virion-like particles (VLPs) in tissue culture supernatants and in whole-cell lysates (WCLs) (BE = GLucVLP/GLucVLP + GLucWCL). Budding-deficient mutant Z-G2A was used as control. Values correspond to four independent replicates. Error bars represent mean +/− SEM. Statistical significance was calculated by analysis of variance (ANOVA) (* *p* < 0.05, ** *p* < 0.002, *** *p* < 0.0002, and **** *p* < 0.00001).

To investigate the mechanism by which these novel DHODH inhibitors exerted anti-mammarenavirus activity, we evaluated the effects of Cmp 1, Cmp 2, Cmp 3, Cmp 4, and Cmp 5 on distinct steps of the LCMV lifecycle (Figure 4). To examine whether DHODH inhibitors affected virus cell entry, a time-of-addition experiment was conducted, in which each compound was added 2 h prior to infection or 2 h pi (Figure 4A). For this experiment, the single-cycle infectious rLCMV∆GPC/ZsG was used to prevent the confounding factors introduced by multiple rounds of infection. All five DHODH inhibitors strongly inhibited virus-mediated ZsG expression regardless of the time of addition, indicating that virus cell entry was not targeted by the tested DHODH inhibitors. F3406, a known inhibitor of LCMV cell entry, was used as the control [47]. To investigate whether the inhibition of virus multiplication by DHODH inhibitors was mediated by reduced viral RNA synthesis, we examined the expression of CAT from a reporter gene driven in the LCMV MG (Figure 4B). Normalized CAT expression was significantly reduced in the presence of all tested DHODH inhibitors, suggesting that the blockade of *de novo* pyrimidine synthesis by DHODH inhibitors interfered with viral RNA synthesis directed by the intracellularly reconstituted LCMV vRNP. Finally, to study the effect of DHODH inhibitors on virion budding, a process directed by the mammarenavirus matrix protein (Z) (Figure 4C), 293T cells were transfected with plasmids expressing LASV-Z-GLuc in the presence of the indicated inhibitors. At 48 h post-transfection, GLuc activity was determined in clarified TCSs and WCLs as surrogates of VLP-associated (Z_VLP_) and intracellular (Z_WCL_) Z levels, respectively. Cmp 1 and 4 had a very modest, but statistically significant, effect on Z budding efficiency as determined by the ratio Z_VLP_:(Z_VLP_+Z_WCL_).

### 3.4. Effect of Pyrimidine Supplementation on the Antiviral Activity of DHODH Inhibitors

**Figure 5:**
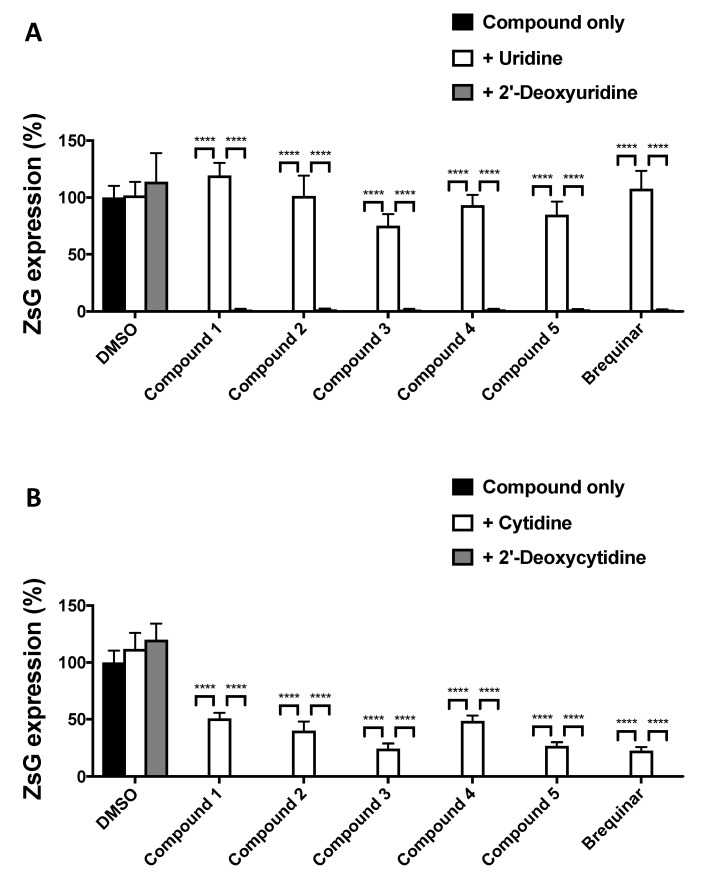
Pyrimidine addition counteracts the antiviral activity of DHODH inhibitors. A549 cells were treated with the indicated DHODH inhibitors (5 µM) and with or without uridine (**A**) or cytidine (**B**) and infected (MOI = 0.01) with rCL-13/ZsG. At 48 h pi, ZsG expression levels were determined. Values correspond to four independent replicates. Error bars represent mean +/− SEM. Statistical significance was calculated by ANOVA (**** *p* < 0.00001).

DHODH is a key enzyme in the *de novo* pyrimidine biosynthesis pathway, and DHODH inhibitors exert their antiviral activity via the depletion of intracellular pyrimidine levels. Accordingly, the antiviral activity of DHODH inhibitors can be reversed by promoting the pyrimidine salvage pathway via supplementation with exogenous pyrimidine ribonucleosides. Treatment of LCMV-infected A549 cells with non-limiting exogenous uridine (Figure 5A) or cytidine (Figure 5B) supplementation reverted completely or partially, respectively, the antiviral effects of DHODH inhibitors. We used 2′-deoxy analogs as negative controls due to their inability to be metabolized for ribonucleotide synthesis.

### 3.5. Contribution of IFN-I to the Mammarenavirus Antiviral Activity of DHODH Inhibitors

**Figure 6:**
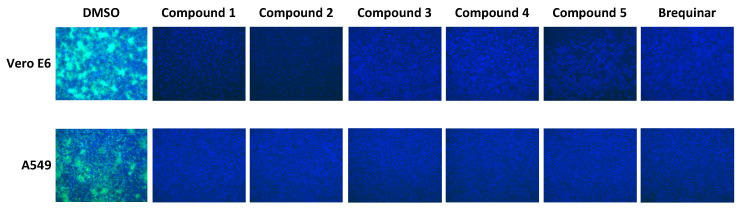
Effect of DHODH inhibitors on LCMV multiplication in interferon (IFN) -deficient (Vero E6) and IFN-competent (A549) cells. IFN-deficient Vero E6 cells and IFN-competent A549 cells were infected (MOI = 0.01) with rCL-13/ZsG in the presence of the indicated DHODH inhibitors (5 µM). At 48 h pi, cells were fixed and stained with DAPI. ZsG-expressing cells were detected by epifluorescence.

**Figure 7:**
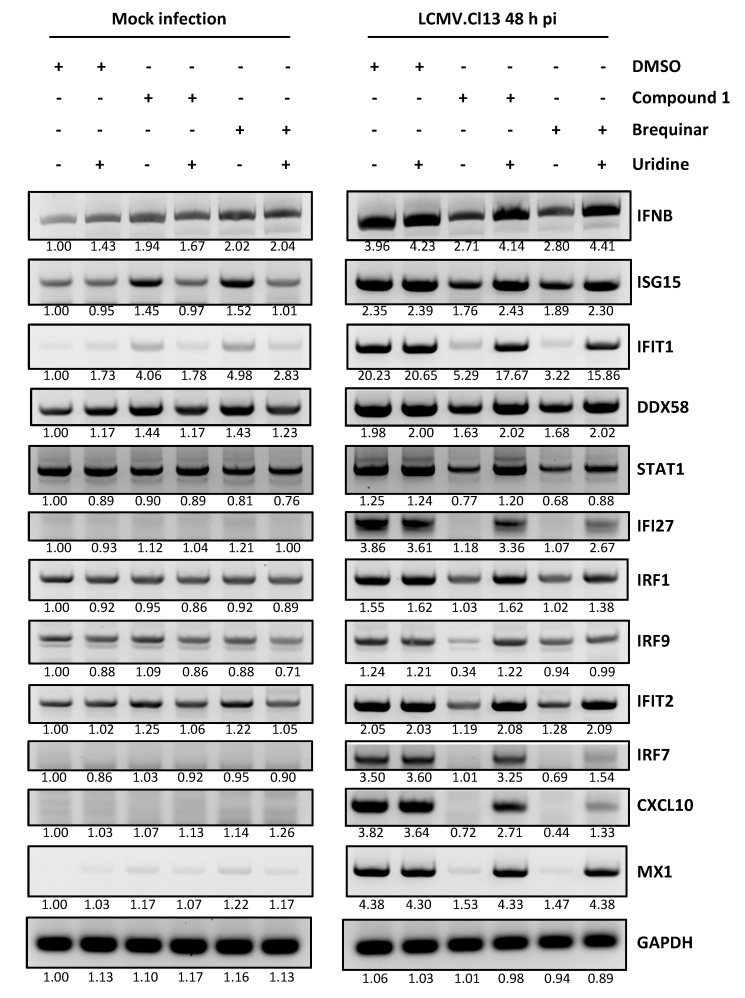
Limited role of pyrimidine depletion-mediated IFN-stimulated genes (ISG) expression on the antiviral activity against LCMV. A549 cells were treated with the indicated DHODH inhibitors (5 µM) and uridine (100 µM), and then incubated for 48 h with or without rCL-13 infection. Total cellular RNA was purified and equal amounts (3.5 µg) were used in reverse transcription reactions using random hexamer primers to generate cDNAs that were used in PCR reactions with specific primers to amplify the cDNAs of indicated genes. PCR products were resolved by agarose gel electrophoresis and visualized by ethidium bromide staining. The number below each band indicates the normalized fold change of band intensity compared to mock-infected and vehicle-treated control cells. Each band intensity was normalized to the corresponding glyceraldehyde 3-phosphate dehydrogenase (GAPDH) band intensity.

Type I and Type III IFN responses could be enhanced by pyrimidine biosynthesis inhibition, which can further contribute to reduced virus multiplication [48]. However, we previously demonstrated that pyrimidine biosynthesis inhibitors exhibited similar antiviral anti-LCMV activity in IFN-competent (A549) and IFN-deficient (Vero E6) cells [25]. Consistent with our previous findings, all five DHODH inhibitors had a similarly potent anti-LCMV activity in both IFN-competent (A549) and IFN-deficient (Vero E6) cells (Figure 6). Some pyrimidine biosynthesis inhibitors have been shown to promote an IFN-independent antiviral cellular state [28,49]. To examine this issue, we selected Cmp 1 as a representative member of the newly tested DHODH inhibitors and used brequinar as a positive control to examine their effects on the expression of ISGs previously shown to be upregulated by pyrimidine biosynthesis inhibitors in an IFN-independent manner (Figure 7). In non-infected A549 cells, ISG15 and IFIT1 were moderately upregulated by treatment with Cmp 1 or brequinar, and the increased expression of ISG15 and IFIT1 was reversed by uridine supplementation (Figure 7, left panel). Neither Cmp 1 nor brequinar treatment significantly affected the expression of IFNB, indicating that upregulated ISG15 and IFIT1 expression was IFNB-independent. LCMV infection appreciably upregulated the messenger RNA (mRNA) levels of IFNB and all of the tested ISGs (Figure 7, right panel). Treatment with Cmp 1 or brequinar significantly decreased the mRNA levels of IFNB and ISGs in LCMV-infected cells, an effect that was reversed by uridine supplementation.

### 3.6. Assessment of Cmp 4 (IM90838) Anti-LCMV Activity In Vivo

**Figure 8:**
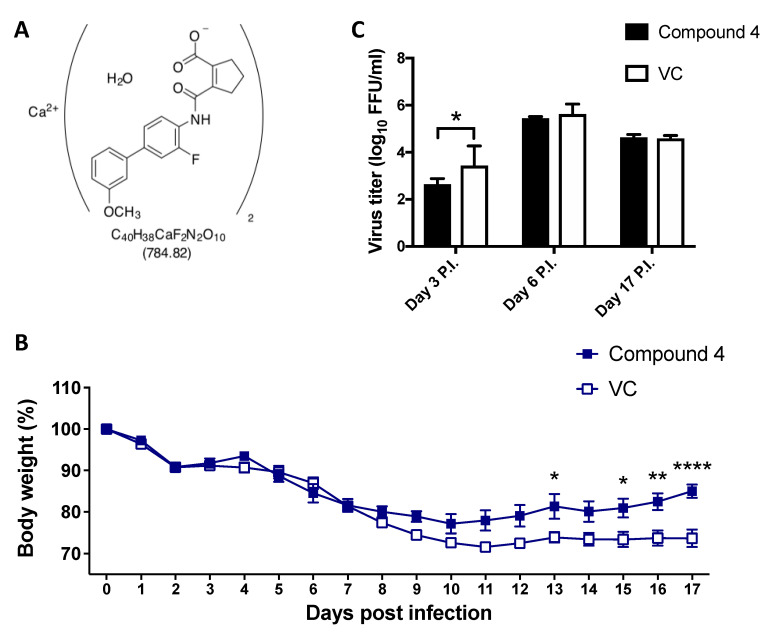
*In vivo* antiviral activity of Cmp 4 against LCMV. (**A**) Structural formula of Cmp 4 (IMU90838, also known as vidofludimus calcium). (**B** and **C**) C57BL/6 mice (*n* = 4) were orally treated with Cmp 4 (150 mg/kg/d) for 17 days or with VC. Both groups of mice were infected (IV) with 2 × 10^6^ pfu of CL-13. Body weight changes (**B**) and serum virus titers (**C**) were determined at the indicated time points. Statistical significance was calculated by ANOVA (* *p* < 0.05, ** *p* < 0.002, and **** *p* < 0.00001).

To assess the antiviral activity *in vivo* of the novel DHODH inhibitors, we used the C57BL/6 mouse model of infection with the immunosuppressive CL-13 variant of the Armstrong strain of LCMV [50]. In this model, IV infection of adult C57BL/6 mice with a high dose (2 × 10^6^ pfu) of CL-13 resulted in transient weight loss and the establishment of viral persistence lasting over 90 days. We selected Cmp 4 (Figure 8A) for this experiment because it is currently under testing in phase 2 clinical trials, with more detailed *in vivo* data available, including toxicology and pharmacology. Following infection, we observed less body weight loss and faster recovery (Figure 8B) in mice treated with Cmp 4 than in the vehicle control group; but, treatment with Comp 4 did not cause a significant reduction in viral load (Figure 8C).

### 3.7. Effect of Inhibiting the Uridine/Cytidine Kinase 2 (UCK2) on the Antiviral Activity of Cmp 4 in the Presence of Unlimited Uridine Supply

**Figure 9:**
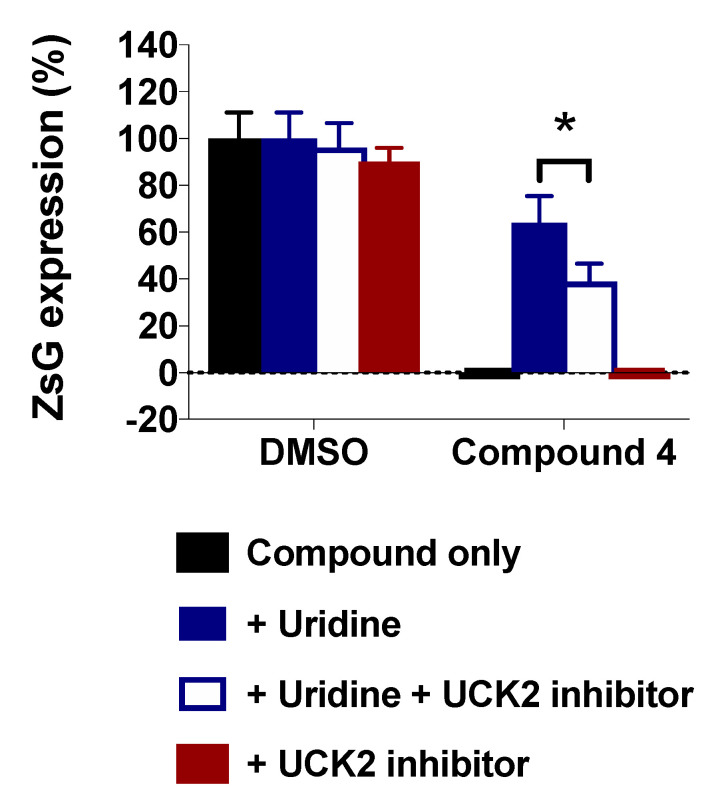
A549 cells were treated with Cmp 4 (5 µM) and with or without uridine or UCK2 inhibitor and infected (MOI = 0.01) with rCL-13/ZsG (six replicates). At 48 h pi, ZsG expression levels were determined. Error bars represent mean +/− SEM. Statistical significance was calculated by ANOVA (* *p* < 0.05).

Several DHODH inhibitors with potent antiviral activity in cultured cells have been shown to be ineffective *in vivo* due to the efficient salvage of exogenous uridine, which could have accounted for the lack of Cmp 4 antiviral efficacy in the mouse model of CL-13 infection. The requirement of UCK2 for the pyrimidine nucleotide salvage pathway led us to examine whether the inhibition of UCK2 enhanced the antiviral activity of the DHODH inhibitor Cmp 4 under conditions of an unlimited uridine supply. For this, we examined the effect of UCK2 inhibitor 20874830 in the pyrimidine supplementation-mediated reversion of the antiviral activity of Cmp 4 (Figure 9). Consistent with our previous results (Figure 5A), supplementation with exogenous uridine reverted the Cmp 4-induced antiviral effect, and treatment with UCK2 inhibitor 20874830 resulted in partial recovery of the antiviral effect of Cmp 4.

## 4. Discussion

In this study, a series of novel DHODH inhibitors were evaluated for their antiviral activities against three mammarenaviruses. The tested DHODH inhibitors exhibited broad-spectrum anti-mammarenavirus activity as they inhibited the multiplication of LCMV, LASV, and JUNV with high SI values in cultured cells. Our results also provide evidence that these DHODH inhibitors do not affect cell entry or budding, but rather the inhibition of RNA synthesis mediated by the vRNP (Figure 4B). This finding is consistent with the well-established effect of DHODH inhibitors on cellular pyrimidine nucleoside pools. This mechanism of action was further supported by the results from pyrimidine supplementation experiments (Figure 5), showing that uridine supplementation reversed the antiviral activity associated with pyrimidine depletion caused by treatment with DHODH inhibitors. Unexpectedly, supplementation with exogenous cytidine showed only partial reversion of the DHODH-mediated antiviral activity (Figure 5B), which might reflect differences in the activity of enzymes in each uridine or cytidine salvage pathway. Thus, maintaining pyrimidine nucleoside pools is likely to be essential for virus propagation, suggesting that pyrimidine depletion is a key mechanism for antiviral effects mediated by DHODH inhibitors.

The contribution of IFN-I to the antiviral effects mediated by inhibitors of pyrimidine biosynthesis remains controversial. Pyrimidine biosynthesis inhibitors promote enhanced IFN-I-mediated innate immune responses contributing to the inhibition of the multiplication of several viruses, including measles virus, chikungunya virus, and West Nile virus [48]. However, pyrimidine depletion was shown to trigger IFN-I-independent expression of ISGs that resulted in cellular antiviral states that inhibited the replication of vesicular stomatitis Indiana virus [49]. In addition, the IFN-independent expression of IRF1 contributed to the antiviral activity of a DHODH inhibitor (SW835) against Ebola virus (EBOV) [28]. For mammarenaviruses, different pyrimidine biosynthesis inhibitors exhibited similar antiviral activities in both IFN-competent and IFN-deficient cells [25]. Similarly, the DHODH inhibitors evaluated in this study are antivirally active in an IFN-independent way against mammarenaviruses (Figure 6). We found that treatment with DHODH inhibitors moderately increased the mRNA levels of ISG15 and IFIT1 in uninfected cells, whereas other ISGs and IFNB were not affected (Figure 7, left panel). Consistent with previous findings [51], LCMV infection increased the transcription of IFNB, which was suppressed by treatment with DHODH inhibitors. Inhibitors of pyrimidine biosynthesis did not affect ISG expression levels in IFN-I-deficient Vero E6 cells (data not shown). These findings suggest that the pyrimidine depletion-induced anti-mammarenavirus effect is IFN-I independent and that ISGs have a minimal contribution to this antiviral effect.

Studies testing the *in vivo* efficacy of pyrimidine biosynthesis inhibitors as antiviral drug candidates have had only moderate success. In mice exposed to a typically lethal dose of influenza A virus, treatment with the DHODH inhibitor FA-613 increased survival [52]. Likewise, intranasal treatment with leflunomide’s active metabolite, A77-1726, reduced the pathophysiological signs associated with human respiratory syncytial virus (HRSV) infection in laboratory mice, but this clinical improvement was not associated with a reduction in lung viral load [53]. In this study, we found that CL-13-infected mice treated with Cmp 4 had less body weight loss and showed a faster recovery (Figure 8). However, treatment with Cmp 4 did not reduce viral load. The pyrimidine salvage pathway can provide LCMV-infected cells with pyrimidine pools that could counteract the effect of DHODH inhibitors. Therefore, targeting the pyrimidine salvage pathway could be a strategy to enhance the antiviral effect of pyrimidine biosynthesis inhibitors *in vivo*. The feasibility of this approach is supported by knockdown mediated by short hairpin RNA (shRNA) and CRISPR-Cas9-mediated deletion of UCK2, a key enzyme of the pyrimidine salvage pathway that sensitized cells to GSK983, a known DHODH inhibitor [54]. Consistent with this observation, we found that co-treatment with Cmp 4 and UCK2 inhibitor 20874830 partially prevented the uridine supplementation-mediated reversion of the antiviral effect exerted by Cmp 4 in cultured cells (Figure 9). Unfortunately, we were unable to test the *in vivo* efficacy of combination therapy with DHODH and UCK2 inhibitors due to a limited supply of UCK2 inhibitor 20874830.

## 5. Conclusions

A series of novel DHODH inhibitors exhibited strong broad-spectrum antiviral activity against mammarenaviruses *in vitro*. The anti-mammarenavirus activity of the tested DHODH inhibitors was mediated by pyrimidine depletion, which negatively impacted viral RNA synthesis and was independent of the IFN-I response. However, the IFN-I independent induction of some ISGs might have contributed to the anti-mammarenaviral activity of the tested DHODH inhibitors.
